# Quality of life after the initiation of dialysis or maximal conservative management in elderly patients: a longitudinal analysis of the Geriatric assessment in OLder patients starting Dialysis (GOLD) study

**DOI:** 10.1186/s12882-019-1268-3

**Published:** 2019-03-29

**Authors:** I. N. van Loon, N. A. Goto, F. T. J. Boereboom, M. C. Verhaar, M. L. Bots, M. E. Hamaker

**Affiliations:** 1 Dianet Dialysis Center, Utrecht, The Netherlands; 2 Department of Nephrology and Hypertension, University Medical Center Utrecht, Utrecht, The Netherlands; 3 Department of Internal Medicine, Diakonessnhuis Utrecht, Utrecht, The Netherlands; 4 Department of Geriatrics, University Medical Center Utrecht, Utrecht, The Netherlands; 5 Julius Center for Health Sciences and Primary Care, University Medical Center Utrecht, Utrecht, The Netherlands; 6 Department of Geriatrics, Diakonessenhuis Utrecht, Utrecht, The Netherlands

**Keywords:** Quality of life, Dialysis, Maximal conservative care, Geriatric assessment

## Abstract

**Background:**

Maximal conservative management (MCM) may be an appropriate alternative option for dialysis in some elderly patients with end-stage kidney disease (ESKD). Evidence about the impact of dialysis or MCM on quality of life (QoL) in older patients is sparse. In the GOLD (Geriatric assessment in OLder patients starting Dialysis) Study the trajectory of QoL was assessed in patients starting dialysis or MCM.

**Methods:**

Patients ≥65 years old were included just prior to dialysis initiation or after decision for MCM. Baseline data included demographics, frailty as measured with a geriatric assessment, comorbidity (CIRS-G) and QoL, measured with the EQ-5D-3 L (EQ-5D Index and overall self-rated health). Six months follow-up data included QoL, hospitalizations and mortality. Change of QoL was assed with paired t-tests. Cox-regression was used to assess survival of MCM and dialysis patients.

**Results:**

The cohort comprised 192 dialysis and 89 MCM patients. The MCM patients were older (mean age 82 ± 6 vs. 75 ± 7 years, *p* <  0.01) and mean kidney function was better (eGFR 11.5 ± 4.0 vs. 8.0 ± 2.9 ml/min/1.73m^2^, *p* <  0.01). Baseline QoL did not differ significantly between the groups. After six months, EQ-5D Index did not improve significantly in the dialysis group with mean ± standard error (SE) 0.026 ± 0.014 (*p* = 0.10; not clinically relevant), but a small but clinically relevant decline was seen in the conservative group: 0.047 ± 0.022 (*p* < 0.01; between group difference *p* < 0.01). Hospitalization occurred in 50% of dialysis patients vs. 24% of conservative patients (*p* < 0.01). In patients over 80 years old, no survival benefit could be found for dialysis patients starting dialysis vs. MCM.

**Conclusion:**

A small decline of QoL was found for conservative patients, while QoL did not change in dialysis patients. However, hospitalization rate was higher in patients starting dialysis. In patients over 80 years, no survival benefit was found.

**Electronic supplementary material:**

The online version of this article (10.1186/s12882-019-1268-3) contains supplementary material, which is available to authorized users.

## Background

The end-stage kidney disease (ESKD) population is ageing, which has resulted in a growing number of elderly patients starting dialysis [1]. In this population, comorbidity burden is high and functional and cognitive impairment are frequently encountered [2, 3]. Almost half of the octogenarians and nonagenarians die within the first year of dialysis initiation [4, 5]. In older patients with multiple comorbidities, starting dialysis does not seem to prolong life as compared to conservative care [6], but does increase the risk of hospitalisation [7]. For elderly patients, the focus of care has shifted from prolonging life to maximizing quality of life. Consequently, maximal conservative care has become an accepted alternative for patients with ESKD, especially in those who are frail. However, it is difficult to predict how dialysis or forgoing dialysis would impact on a patient’s symptom burden and quality of life. Evidence on this trajectory in patients choosing maximal conservative care is sparse. This leads to insecurity in the process of shared decision-making [8].

In the GOLD (Geriatric assessment in OLder patients starting Dialysis) Study, elderly patients (≥65 years old) were followed in the first six months after the start of renal replacement therapy or the decision for maximal conservative therapy only. Quality of life was assessed at baseline and at six-months follow-up, and mortality and hospitalization data were also collected. The goal of this analysis is to assess quality of life in patients starting dialysis and patients choosing maximal conservative care.

## Methods

### Study design and patient selection

The GOLD Study (Geriatric assessment in OLder patients starting Dialysis) is a multicenter, prospective cohort study assessing the relation between a geriatric assessment and poor outcome in patients with ESKD. Participants were enrolled from 17 different hospitals across the Netherlands In the period August 2014 to September 2017, participants were enrolled from hospitals across the Netherlands (Additional file 1: Table S1). The population consisted of two groups of consecutive patients: patients starting dialysis and patients choosing maximal conservative management. For the dialysis group, patients were included < 3 weeks before and < 2 weeks after the first dialysis session. If dialysis would start > 3 week after the geriatric assessment was performed, patients were excluded from the follow-up. For the maximal conservative management group, patients were included < 3 months after the decision to forgo dialysis had been made *and* if GFR was < 15 ml/min (either estimated with CKD-EPI or measured with 24-h urine creatinine clearance). The decision to forgo dialysis was made after shared-decision making according local practice of the pre-dialysis clinic. If patients had made the decision for conservative therapy, but creatinine clearance was above the cut-off value, they were followed and approached again for enrollment once GFR had fallen below 15 ml/min. Patients were excluded if informed consent was not provided, if they had insufficient understanding of the Dutch language or if they suffered from a terminal non-renal related condition. The study was conducted in accordance with the Declaration of Helsinki. The study was approved by the medical ethics review boards of all participating hospitals and written informed consent was obtained from all patients prior to enrollment.

### Data collection

Baseline demographic data collected from the medical charts and during the baseline assessment included age, sex, educational level and living situation. Other clinical characteristics included cause of kidney failure, blood pressure, body mass index (BMI) and smoking habit. For dialysis patients, type of dialysis and dialysis access were recorded.

For the baseline assessment, including a health related quality of life (HRQOL) questionnaire and a geriatric assessment (GA), participants were either visited at home (on a non-dialysis day for hemodialysis patients) or in the dialysis center, before starting the dialysis session. The assessments were performed by the investigators (IL or NG) or by one of the trained research nurses.

### Health related quality of life

HRQOL was measured using the EuroQol-5D-3 L, which consists of two parts [9]. First, a self-reported 5-item questionnaire addressing the amount of problems experienced in mobility, self-care, usual activities, pain/discomfort and anxiety/depression. Impairment was scored per item, as no, moderate or severe impairment. An impaired health status was defined as ≥1 moderate or severe problem. The domains were subsequently converted into a single summary index, “EQ-5D Index”, by applying a formula that attaches values to each of the levels in each dimension. The index can be calculated by deducting the appropriate weights from 1, the value for full health (i.e. state 11,111). Subsequently, we used the Dutch Tariff for correction of the societal valuation of QoL in The Netherlands [10]. Second, all patients were asked to indicate overall self-rated health on the EQ-5D-3 L visual analogue scale (VAS, scale 0 to 10 where 0 is the worst imaginable health state and 10 the best imaginable health state).

### Geriatric assessment

Frailty was assessed with a geriatric assessment, generally considered the best systematic approach for identification of frailty [11–13]. It focuses on the following domains: (instrumental) activities of daily living, mobility, cognition, mood, nutritional status and comorbidity burden. Comorbidity burden was assessed with the Cumulative Illness Rating Scale-Geriatrics (CIRS-G) and ≥ 2x score 3 or ≥ 1x score 4 was considered a severe comorbidity burden [14]. Patients were considered frail if they had impairments in ≥2 geriatric domains [15]. More detailed information can be found in Additional file 2: Table S2.

### Follow-up

After six months, data on hospitalizations and complications were collected from each center. Mortality data were collected at 6 and 12 months. The patients who were alive after six months were contacted by telephone and the EuroQol-5D-3 L questionnaire was applied again.

### Statistical analysis

Data were summarized using means with standard deviation (SD), medians with interquartile ranges, or proportions when appropriate. Differences between the dialysis patients and conservative patients regarding baseline characteristics and outcomes were assessed using chi-squared tests for dichotomous variables, t-tests for normally distributed continuous variables and non-parametric tests for non-normally distributed continuous variables. For the difference in health related QoL domains at baseline and at follow-up, a logistic regression was subsequently applied, adjusting for age (years) and eGFR (ml/min/1.73m^2^).

The difference in EQ-5D Index and overall self-rated health status between baseline and follow-up was assessed with a paired t-test, the between group differences was assessed with a t-test. The change of the EQ-5D Index and global health between dialysis and conservative patients was assessed with a t-test. A difference of ≥0.03 point of the EQ-5D Index is considered the minimal clinically important difference (MCID) [16]. For global health (EQ-5D VAS) a difference of 0.7 or 0.8 is considered the MCID [17, 18]. Follow-up outcomes of the EQ-5D Index were defined as: improved (≥0.03 point improvement, or having received a kidney transplant), equal (change < 0.03 point), deterioration (≥0.03 point decline) or death. Analysis of baseline EQ-5D Index and VAS and change over time was repeated with sensitivity analyses, which excluded the dialysis patients on the waiting list and patients who received a kidney transplant during follow-up.

As we hypothesized that most dialysis patients may have a lower eGFR compared to conservative patients (who were included with a GFR < 15 ml/min), a subgroup analysis was performed for patients with an eGFR < 10 ml/min/1.73m^2^ and patients with an eGFR ≥10 ml/min/1.73m^2^. In addition, a subgroup analysis was performed for patients aged < 80 years and ≥ 80 years old, as QoL becomes even more relevant in this group as dialysis does not seem to prolong life in this population [19].

Mortality rates between dialysis and conservative patients were assessed with a log-rank test. As 6-month mortality rate was low, an extended multivariate analysis was performed for 12-month mortality rate. A Cox-regression model was used, adjusting for age (years), comorbidity burden and eGFR category.

A two-tailed *p* < 0.05 was considered statistically significant. Data analysis was performed with SPSS version 22 software [15].

## Results

### Baseline characteristics

A total of 281 patients were included in the GOLD Study, of whom 192 started dialysis (23% PD) and 89 choose maximal conservative therapy. Another 42 patients were screened, but excluded because of reasons mentioned in the Flowchart (Additional file 3: Figure S1). The baseline characteristics of the dialysis patients and conservative patients are summarized in Table 1. Conservative patients were older (mean ± SD 82 ± 6 vs. 75 ± 7, *p* < 0.01) and more likely to live alone (56% vs. 42%, *p* = 0.03). In addition, kidney function of conservative patients was more preserved; in 61% of patients choosing conservative care eGFR was > 10 ml/min/1.73m^2^, compared to 22% of dialysis patients (*p* < 0.01). Haemoglobin and albumin levels were significantly higher in conservative patients (Table 1).

**Table 1:** Baseline characteristics

	*Dialysis (n = 192)*	*Conservative (n = 89)*	*P value*
*Demographics*			
*Age, years, mean ± SD*	*75 ± 7*	*82 ± 6*	*< 0.01*
*Gender (% male)*	*128 (67%)*	*50 (56%)*	*0.07*
*Clinical parameters*			
*Cause of kidney failure (%)*			*0.40*
*Renal vascular*	*96 (50%)*	*45 (51%)*	
*Diabetes*	*31 (16%)*	*17 (19%)*	
*Nephritis*	*12 (6%)*	*5 (6%)*	
*Other*	*54 (28%)*	*25 (28%)*	
*Dialysis modality (% PD)*	*44 (23%)*	*–*	
*Access: Central venous line (% of HD)*	*73 (38%)*	*–*	
*BMI (kg/m2), mean (±SD)*	*27 ± 5*	*26 ± 5*	*0.43*
*Systolic blood pressure (mmHg)1*	*150 ± 22*	*151 ± 26*	*0.73*
*Diastolic blood pressure (mmHg)1*	*75 ± 14*	*75 ± 13*	*0.74*
*Smoking (former, now), n (%)*	*148 (77%)*	*62 (70%)*	*0.19*
*Severe comorbidity*^a^	*79 (41%)*	*39 (44%)*	*0.69*
*Frailty*^b^	*148 (77%)*	*78 (88%)*	*0.06*
*Laboratory values* ^c^			
*Hemoglobin (mmol/L)*	*6.4 ± 0.9*	*7.1 ± 0.9*	*< 0.01*
*Albumin (g/L)*	*34 ± 6*	*37 ± 6*	*< 0.01*
*eGFR CKD-EPI in ml/min/1.73 m2*	*8.0 ± 2.9*	*11.5 ± 4.0*	*< 0.01*
*< 10 ml/min/1.73 m2 (%)*	*152 (79%)*	*35 (39%)*	*< 0.01*
*≥10 ml/min/1.73 m2 (%)*	*40 (21%)*	*54 (61%)*	
*Social setting*			
*Living alone*	*81 (42%)*	*50 (56%)*	*0.03*
*Living in a nursing home facility*	*10 (5%)*	*7 (8%)*	*0.36*
*University*	*40 (21%)*	*16 (18%)*	*0.50*
*Polypharmacy*			
*Mean no of drugs ± SD*	*12 ± 5*	*10 ± 3*	*< 0.01*

*Legend: SD standard deviateion, PD peritoneal dialysis, HD Hemodialysis, BMI body mass index,*

*eGFR estimated glomerular filtration rate, 1Measured before dialysis session in dialysis patients*

^a^ *Measured with CIRS-G, severe comorbidity is ≥ 2x socre 3 or ≥ 1x score 4,*

^b^*Measured with the geriatric assessment (see Supplemental material* Table *2**)*

^c^ *Measured before the first dialysis session in dialysis patients*

Frailty was prevalent in 88% of the conservative patients vs. 78% of the dialysis patients (*p* = 0.06). Of all dialysis patients, 26 (13%) were on the waitlist for kidney transplantation.

### Quality of life at baseline

The EQ-5D summary index of conservative patients (mean score ± standard deviation (SD) 0.77 ± 0.21) did not differ significantly from the score of dialysis patients (0.82 ± 0.18 SD, *p* = 0.05); nor did it differ from the score of dialysis patients not on the waiting list (0.81 ± 0.18 SD, *p* = 0.10).

In addition, overall self-rated health (EQ-5D VAS) was comparable at the moment of choosing conservative care and initiating dialysis: 6.3 ± 1.3 for conservative patients vs. 6.3 ± 1.4 in dialysis patients (*p* = 0.91). The latter score was the same for those not on the waiting list.

**Fig. 1:**
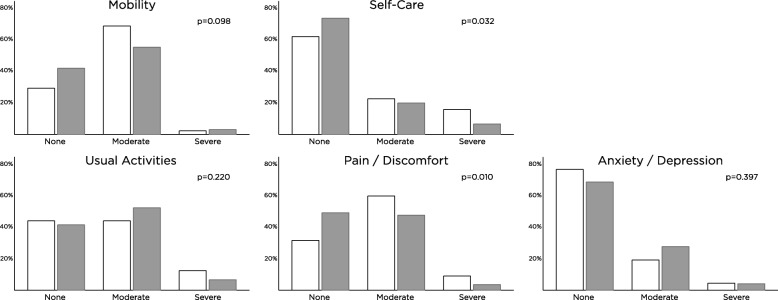
EuroQolD5 Quality of Life at baseline. Legend: white = patients choosing maximal conservative management, grey = dialysis patients

Figure 1 shows the 5 EQ-5D domains at baseline. For the domain pain/discomfort, 69% of conservative patients reported an impaired health status compared to 51% of dialysis patients (*p* < 0.01; Table 2). Anxiety/depression was reported in 31% of dialysis patients vs. 24% of conservative patients (*p* = 0.22). Mobility was impaired in 58% of dialysis patients vs. 71% of conservative patients (*p* = 0.04). After adjusting for age and eGFR, pain/discomfort remained significantly higher in the conservative group compared to dialysis (OR 2.25 [95% CI 1.18–4.30) and anxiety/depression was lower in the conservative group (OR 0.45 [95% CI 0.22–0.92]). There were no significant differences in mobility, usual activities and self-care.

**Table 2:** Percentage of patients with impaired health status^a^

	Dialysis, n (%)	Conservative care, n (%)	*P* value
Baseline			
Mobility	111 (58)	63 (71)	0.04
Self-care	51 (27)	34 (38)	0.05
Usual activities	112 (58)	50 (56)	0.73
Pain/Discomfort	98 (51)	61 (69)	< 0.01
Anxiety/Depression	59 (31)	21 (24)	0.22
Follow-up			
Mobility	95 (55)	57 (78)	< 0.01
Self-care	42 (24)	30 (41)	< 0.01
Usual activities	92 (53)	46 (63)	0.16
Pain/Discomfort	76 (44)	48 (66)	< 0.01
Anxiety/Depression	33 (19)	17 (24)	0.42

^a^Percentage of patients with moderate or severe impairment in the EQ-5D domains. Result of the univariate analysis

### Follow-up

#### Mortality

No patients crossed over from the conservative group to the dialysis group. Transplantation rate was 2% (*n* = 3). Six-months mortality rate differed between the groups, but not significantly: mortality rate in conservative patients was 15% compared to 8% in dialysis patients (*p* = 0.12).

**Fig. 2:**
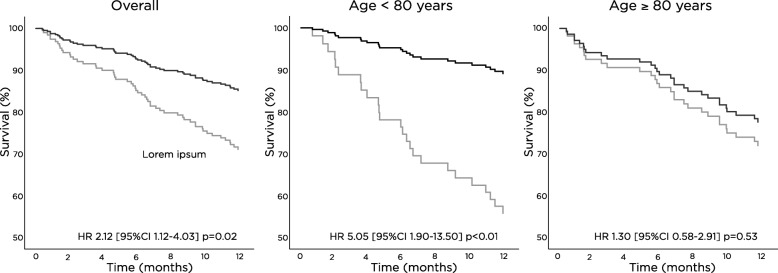
Cox regression conservative therapy vs. dialysis. Legend: Adjusted for age, comorbidity, eGFR

In the extended 12-months analysis, mortality rate in conservative patients was 34% compared to 16% in dialysis patients (*p* = 0.01). Transplantation rate was 4% (*n* = 8). After adjusting for age, comorbidity level and GFR category, hazard ratio (HR) for twelve-month mortality for conservative care vs. dialysis was 2.12 (95% CI 1.12–4.03; Fig. 2). Among patients over 80 years old, conservative care vs. dialysis was not related to mortality (adjusted HR 1.30 [95% CI 0.58–2.91], Fig. 2).

#### Trajectory of quality of life

Follow-up QoL data were available for 98% of dialysis patients and for 92% of conservative patients (Additional file 3: Figure S1). After six months, anxiety/depression decreased in the dialysis group from 31% tot 21% (*p* < 0.01), while in the conservative group this remained stable (24%, Table 2); the other domains did not change significantly. Overall, more impairment was found in the conservative group at follow-up. Mobility impairment (78% conservative vs. 55% dialysis, *p* < 0.01), self-care impairment (41% vs. 24%, *p* < 0.01) and pain/discomfort (66% vs. 44%, *p* < 0.01) were more prevalent in the conservative group. However, when adjusted for age and eGFR no significant differences in EQ-5D domains were found between the two groups (data not shown).

The EQ-5D Index did not change significantly and relevantly in the dialysis group with 0.026 ± 0.014(±SE) (*p* = 0.10), but a small but clinically relevant decline was seen in the conservative group with (0.047 ± 0.022(±SE) (*p* < 0.01; difference between the groups *p* < 0.01)). The sensitivity analysis which excluded patients on the waiting list, showed no significant change either (0.026 ± 0.027(±SE) (*p* = 0.09), between group difference *p* < 0.01).

In the dialysis patients, two-thirds showed a stable or improved EQ-5D score while this occurred in half of the conservative patients (*p* < 0.001, Table 3). The same results were found for older patients (≥80 years) and for patients with a low GFR. However, for patients with a high GFR the differences between the dialysis group and the conservative group were less pronounced and no significant difference could be found (Table 3).

**Table 3:** Trajectory of Quality of life first 6 months (EQ-5D Index)

	Total (n)	Outcome				
		Dead	Decline	No change	Improvement/(Transplantation)	*P* value
*All*						
Conservative, n (%)	83	18 (16)	26 (31)	32 (39)	12 (15)	
Dialysis, n (%)	185	16 (9)	41 (22)	53 (29)	75 (42) (2%)^#^	< 0.001
*Sub analyses*						
*Creatinine clearance*						
eGFR < 10 ml/min/1.73m^2^						0.01
Conservative, n (%)	32	5 (16)	11 (34)	13 (41)	3 (9)	
Dialysis, n (%)	145	13 (9)	28 (19)	42 (29)	62 (43) (1%)^#^	
eGFR ≥10 ml/min/1.73m^2^						0.27
Conservative, n (%)	51	8 (16)	15 (29)	19 (37)	9 (18)	
Dialysis, n (%)	39	3 (8)	12 (31)	11 (28)	13 (33) (2%)^#^	
*Age*						
< 80 years						0.08
Conservative, n (%)	21	4 (19)	6 (29)	8 (38)	3 (14)	
Dialysis, n (%)	132	10 (8)	31 (24)	37 (28)	54 (41) (2%)^#^	
≥ 80 years						0.02
Conservative, n (%)	62	9 (15)	20 (32)	24 (39)	9 (15)	
Dialysis, n (%)	53	6 (11)	10 (19)	16 (30)	21 (40)	

^#^Including transplantation (total *n* = 3)

Overall self-rated health score of dialysis patients improved with 0.3 ± 1.4 points (*p* < 0.01), while QoL score of conservative patients decreased with 0.4 ± 1.1 points (*p* < 0.01; difference between the groups *p* < 0.01), but this was not clinically relevant. The same results were found when patients on the waiting list were excluded (0.3 ± 1.1points (*p* = 0.02, between group difference *p* < 0.01).

### Other complications

Hospitalizations (≥1 in six months) occurred in 50% of dialysis patients and in 24% of conservative patients (*p* < 0.01). Among hospitalized patients, median number of admissions was 1 [range 1–5] for dialysis patients and 1 [range 1–4] for conservative patients (*p* = 0.27). Median number of admission days was 7 [interquartile range, IQR, 3–15] for dialysis patients and 4 [IQR 2–12] for conservative patients (*p* = 0.22). Three dialysis patients and one conservative patient moved to a nursing home facility and one conservative patient was admitted to a hospice. Six dialysis patients withdrew from dialysis due to poor overall quality of life or severe complications.

## Discussion

In this analysis, quality of life was compared between prospective cohorts of older patients starting dialysis and older patients choosing conservative care. Several conclusions can be drawn from this study. First, patients starting dialysis experience less pain/discomfort compared to patients choosing conservative care, but overall QoL is comparable. Second, over time, QoL remained stable in the dialysis group, while a small decline of QoL was seen in the conservative group. Third, significantly more dialysis patients were hospitalized at least once compared to conservative patients, despite the conservative patients being older. And finally, 12-month survival in patients over 80 years old is not significantly longer in patients starting dialysis compared to patients choosing conservative care.

So far, only few studies prospectively compared the trajectory of QoL of conservative care patients with dialysis patients or patients preparing for dialysis [20–22]. An Australian study assessed QoL by means of the Short-Form 36 Survey (SF-36) and compared the QoL trajectory of 140 pre-dialysis patients with 30 conservative patients (mean GFR 16 ml/min) [20]. Baseline QoL was worse in conservative patients compared to patients planned for dialysis, but change over a 12-months follow-up period was comparable between the groups. Two other studies using the SF-36 found QoL to be stable over a two-year period in conservative patients and patients who were planned for or started dialysis [21, 22]. All studies were small, including 30–68 patients who were conservatively managed, and none of the studies focused specifically on elderly dialysis patients. Our study expands the prior observations, with a larger cohort of conservative patients and a very high follow-up rate, showing that EQ-5D QoL slightly decreases after the decision for conservative care has been made, but remains stable in elderly incident dialysis patients over a six months period. In addition, while overall self-rated quality of life was comparable between the groups at baseline, the mean score of 6.3 on a scale from 0 [poor]-10 [good]) is much worse compared to community dwelling elderly (7.9 ± 2.3) [9].

Another way of looking at quality of life is looking at admission rate and complications. In our study, half of the dialysis patients were hospitalized compared to one out of four of the conservative patients.

In a UK cohort with a better survival rate for patients choosing dialysis (*n* = 173) compared to conservative care (*n* = 29), the number of hospital-free days was comparable between the groups, when both hospitalization and dialysis days were taken into account [23]. This suggests that overall the number of days at home is more comparable between the two treatment modalities than would be expected based on survival only.

In addition, in the dialysis patients in our cohort, 6 out of 15 deaths (40%) in the first six months after dialysis initiation occurred after withdrawal of dialysis, indicating poor tolerance of the therapy or dissatisfaction with QoL. In a large cohort of more than 12.000 dialysis patients and 800 conservative patients, that focused on the final months before death, conservative patients had significantly less hospitalizations (OR 0.40 [95% CI 0.34–0.46]), less invasive procedures (OR 0.15[95% CI 0.10–0.22]), more palliative care consultation (OR 4.19 [95% CI 3.58–4.90]), more hospice deaths (OR 3.32 [95% CI 2.83–3.89]) and less hospital deaths (OR 0.78 [95% CI 0.74–0.82]) [24]. More qualitatively good days at home and a better anticipation to death in the last phase, suggest that some aspects of QoL may be better in conservative patients compared to dialysis patients. Although it is difficult to capture this in (health related) QoL assessments, it is valuable information to discuss with patients.

There are several limitations in the comparison of QoL between dialysis patients and conservative patients. First there may be a significant lead-time bias, i.e. conservative patients were likely to be included earlier in their illness trajectory, as reflected by higher GFR level. This factor may have led to a relatively higher EQ-5D score for the conservative patients, compared with the score they would have had at a GFR level of < 10 ml/min. We corrected for this by dividing the cohort into two groups (eGFR under and above 10 ml/min/1.73m^2^) and performed the major analyses in two groups.

A second limitation is that we have included conservative patients just after the decision for conservative management had been made, provided that GFR fell below 15 ml/min. However, in clinical practice, the decision may have been pending for a while (even years) in some patients and patients may still switch to dialysis therapy further in their illness trajectory. This should be borne in mind when interpreting these results, as this may have contributed to overestimation of QoL of conservative patients as well.

In addition, follow-up period is fairly short and we only had two QoL measurements. Impact of hospitalization, which in the elderly often involves deterioration of functional abilities, and thus QoL, without full recovery [25], could have been missed by our assessment. However, although the transition to dialysis can have a large impact on elderly patients, our results show that in two-thirds of patients QoL improves or remains stable afterwards.

Finally, QoL measurements have been developed based on perceived quality of health in the general population. Although the EQ-5D has been widely used among CKD patients [26], other values may apply in the conservative care population. When discussing conservative care it is important to find out which values and aspects of physical, cognitive and psychosocial domains matter most to the patient.

The EQ-5D does not only capture objective aspects of quality of life, but also includes a subjective or self-rated part, the visual analogue score. In our cohort, the between group difference of overall self-rated quality of life at follow-up was 0.7 points. This number is considered the minimum clinically important difference (MCID) in cancer [18], although others found 0.8 points was a better cut-off value in COPD [17]. No MCID for ESKD exists. As the within group differences were small and the between group difference was borderline relevant, we considered the change not clinically relevant overall. But one could argue that conservative patients have also some lower self-rated QoL over time, when interpreting the results more strictly.

## Conclusions

Based on the findings from this study, the trajectory of QoL seems slightly better in elderly patients (≥65 years old) starting dialysis compared to patients choosing conservative care in the first six months of follow-up. Two-thirds of dialysis patients remain in a stable of better QoL, while this occurs in half of the conservative patients. Overall, mean EQ-5D Index did not improve in the dialysis group, and a small decline was seen in the conservative group. On the other hand, twice as many dialysis patients were hospitalized within this period. In addition, in patients over 80 years old, no survival benefit could be found for dialysis patients starting dialysis vs. patients choosing conservative care. Overall, in octogenarians and nonagenarians, conservative care may be a good alternative for dialysis.

## Additional files

Additional file 1: **Table S1**. List of participating centers across The Netherlands. (DOC 22 kb)

Additional file 2: **Table S2**. Geriatric assessment. (DOC 43 kb)

Additional file 3: **Figure S1**. Patient flow diagram. (DOC 71 kb)

## Abbreviations

CI: Confidence interval
CIRS-G: Cumulative Illness Rating Scale-Geriatrics
CKD-EPI: Chronic Kidney Disease Epidemiology Collaboration
COPD: Chronic Obstructive Pulmonary Disease
eGFR: estimated glomerular filtration rate
ESKD: End-stage kidney disease
GA: Geriatric assessment
GFR: Glomerular filtration rate
GOLD: Geriatric assessment in OLder patients starting Dialysis
HR: Hazard ratio
HRQOL: Health related quality of life
IQR: Interquartile range
MCID: Minimal clinically important difference
MCM: Maximal conservative management
OR: Odds ratio
QoL: Quality of Life
SD: Standard deviation
SF-36: Short-Form 36 Survey
