# Supplementary material for: Quality of life after the initiation of dialysis or maximal conservative management in elderly patients: a longitudinal analysis of the Geriatric assessment in OLder patients starting Dialysis (GOLD) study
Source: BMC Nephrol. 2019 Mar 29;20:108. doi: 10.1186/s12882-019-1268-3 (PMC6440027; doi:10.1186/s12882-019-1268-3)
Supplement: Supplementary file 1 — Table S1. List of participating centers across The Netherlands. (DOC 22 kb) [file 12882_2019_1268_MOESM1_ESM.doc]

**Supplemental material Table 1. List of participating centers across The Netherlands**

Albert Schweitzer Hospital, Dordrecht; Amsterdam University Hospital, Amsterdam; Bernhoven Hospital, Uden, Diakonessenhuis, Utrecht; Groene Hart Hospital, Gouda; Jeroen Bosch Hospital, ‘s Hertogenbosch; Spaarne Gasthuis, Hoofddorp, Haarlem; Maasstad Hospital, Rotterdam, St Antonius Hospital, Nieuwegein; St Elisabeth Hospital, Tilburg; Franciscus Gasthuis & Vlietland hospital, Rotterdam, Schiedam; Ter Gooi Hospital, Hilversum; University Medical Center Utrecht, Utrecht; Zaans Medical Center, Zaandam; Gelderse Vallei Hospital, Ede.
