# Supplementary material for: Quality of life after the initiation of dialysis or maximal conservative management in elderly patients: a longitudinal analysis of the Geriatric assessment in OLder patients starting Dialysis (GOLD) study
Source: BMC Nephrol. 2019 Mar 29;20:108. doi: 10.1186/s12882-019-1268-3 (PMC6440027; doi:10.1186/s12882-019-1268-3)
Supplement: Supplementary file 2 — Table S2. Geriatric assessment. (DOC 43 kb) [file 12882_2019_1268_MOESM2_ESM.doc]

| **Supplemental material Table 2.** Geriatric assessment   | Domain | Test | Category | Range | Cut-off | Source | | --- | --- | --- | --- | --- | --- | | ADL | Katz-scale |  | 1-6 | (≥1) | Patient | | iADL | Lawton & Brody |  | 1-7 | (≥1) | Patient | | Mobility | Timed up and go^&^ | Severe |  | (> 20 s) | Patient | | Cognitive impairment | MMSE |  | 0-30 | (<25) | Patient | |  | Clock |  | 0-14 | (≤10) |  | |  | ECR |  | 0-16 | (<14) |  | |  | Fluency |  | 0-40 | (<5^th^ percentile)^ |  | | Depressive symptoms | GDS | *No impairment*  *Mild symptoms* | 0-15 | (<5)  (5-10) | Patient | |  |  | Severe symptoms |  | (>10) |  | | Malnutrition | MNA | *Malnutrition* | 0-30 | 0-17) | Patient | | Comorbidities | CIRS-G | Severe^#^ |  | | Chart | | Frailty | | | | | | | Geriatric Assessment% | | ≥ 2 Geriatric Impairments | 0-7% | (≥2) |  | | *Legend*  *% Sum of impairment in (i)ADL, severe mobility impairment, impairment in ≥ cognitive domain, severe depressive symptoms, malnutrition, severe comorbidity score ^&^The average of three measurements was recorded; Immobility was scored as severely impaired ^corrected for age and education level ^#^CIRS-G ≥ 2x score 3 or ≥ 1x score 4; renal comorbidity excluded*  *(i)ADL (instrumental) activities of daily living MMSE Mini Mental State Examination GDS Geriatric Depression Scale ECR Enhanced cued recall MNA Mini Nutritional Assessment CIRS-G Cumulative Illness Rating Scale Geriatrics* | | | | | | |
| --- | --- | --- | --- | --- | --- | --- | --- | --- | --- | --- | --- | --- | --- | --- | --- | --- | --- | --- | --- | --- | --- | --- | --- | --- | --- | --- | --- | --- | --- | --- | --- | --- | --- | --- | --- | --- | --- | --- | --- | --- | --- | --- | --- | --- | --- | --- | --- | --- | --- | --- | --- | --- | --- | --- | --- | --- | --- | --- | --- | --- | --- | --- | --- | --- | --- | --- | --- | --- | --- | --- | --- | --- | --- | --- | --- | --- | --- | --- | --- | --- | --- | --- | --- | --- | --- | --- | --- | --- | --- | --- |
