# Supplementary material for: Quality of life after the initiation of dialysis or maximal conservative management in elderly patients: a longitudinal analysis of the Geriatric assessment in OLder patients starting Dialysis (GOLD) study
Source: BMC Nephrol. 2019 Mar 29;20:108. doi: 10.1186/s12882-019-1268-3 (PMC6440027; doi:10.1186/s12882-019-1268-3)
Supplement: Supplementary file 3 — Figure S1. Patient flow diagram. (DOC 71 kb) [file 12882_2019_1268_MOESM3_ESM.doc]

**Supplemental material Figure 1. Patient flow diagram**
